# Medical Swindlers

**Published:** 1885-04

**Authors:** 


### MEDICAL SWINDLERS.

The managers of the Journal heartily endorse the sentiments expressed in the following clipping from *A New Idea*, but would add to the list that class of swindlers who publish books tending to poison the mind and degrade the understanding.

These books, which usually pretend to discourse on the "Science of life," or the "philosophy of marriage," are without exception, pernicious in their teachings and obscene in their language.

"We would like to make a department in our paper devoted to exposing that class of medical swindles which originates and is carried on most largely in our great cities, wherein certain frauds get their living by using the United States mails for distributing so-called free prescriptions after the manner of the "clergyman whose sands of live have nearly run out." These old so-called free prescription swindles are cropping out everywhere under new names and new titles, and we would take much pains to expose them wherever the facts can be obtained. The method employed by the swindlers is to mail a printed prescription with directions for preparation, which prescription is so worded that it either contains drugs which do not exist, or else drugs not found in commerce, so that the person receiving it is compelled to send to the party advertising in order to obtain the medicine, and of course that is where the trick comes in, the price being may be ten times the value of the medicine."

